# Existing Building Renovation: A Review of Barriers to Economic and Environmental Benefits

**DOI:** 10.3390/ijerph20054058

**Published:** 2023-02-24

**Authors:** Haolan Liao, Rong Ren, Lu Li

**Affiliations:** 1 School of Economics, Shanghai University, 99 Shangda Road, Baoshan District, Shanghai 200444, China; 2 School of Architecture and Urban-Rural Planning, Fuzhou University, Fuzhou 350025, China; 3 College of Environmental Science Engineering, Hunan University, Changsha 410082, China

**Keywords:** carbon footprints, embedded carbon, building renovation, life cycle assessment

## Abstract

The renovation of old buildings provides an important approach to energy saving and emission reduction with low economic costs. The current important issue remains how to determine the optimal cost-effective technical path for a specific project, although there are a large number of retrofit technologies to choose from. Based on a systematic perspective, this paper conducts a quantitative analysis of the environmental and economic benefits of building renovation, and compares and studies the role and challenges of different countries in the process of recycling waste building materials and technological innovation to extend the life of buildings. Through the use of VOSviewer, 1402 papers from the Web of Science core collection database were visualized, analyzed, and deduced, and the research context and development trends of architectural renovation were sorted out and presented. Finally, this article discusses the status and application process of existing building renovation technologies, including the current obstacles that need to be resolved. It puts forward a vision for the future development of building renovation, emphasizing that top-down guidance is essential to future carbon neutral goals.

## 1. Introduction

Increased extreme weather and climate events caused by climate changes worldwide pose an increasing risk to human society [1,2,3]. After the Copenhagen climate change conference, especially the signing of the Paris Agreement, climate change has become an international consensus. Scientists have conducted scenario analysis on the long-term relationship between greenhouse gas emissions and temperature rises, and have set a goal and road map for controlling the temperature rise within 2 ${}^{{}^{\circ}}$C and striving to achieve 1.5 ${}^{{}^{\circ}}$C in the 21st century [4,5,6,7]. Many countries and regions have also successively committed to independent emission reduction goals and formulated the road map and support policies to achieve these goals, governments, enterprises, social organizations, and individuals are taking positive actions to deal with climate change [8,9,10]. In the past 20 years, greenhouse gas emissions have been one of the main causes of the climate crisis. One of the main contributors to greenhouse gas emissions and energy consumption is the construction industry [11].

Buildings account for 40% and 33% of global energy use and greenhouse gas emissions [12,13], and the built environment has a great impact on climate change [14,15,16]. Among them, the construction of new buildings accounts for a quarter of global greenhouse gas emissions, and the heating of existing building stock accounts for one third of emissions [17]. Buildings are already the largest energy consumers in the world, but they will continue to be a source of increasing energy demand in the future. Driven by economic growth and population growth, the final energy consumption of the construction industry doubled between 1971 and 2010, and global construction energy demand is expected to increase by another 30% by 2035 according to current policies [18]. The current research focus is still mainly on new buildings, but existing buildings provide the greatest potential for greenhouse gas emission reduction, and the field of renovation will receive more and more attention [19].

There are some past civilizations that collapsed due to their inability to deal with the environmental problems they caused, such as the Mayans, Easter Island people, and the Anasazi civilization [20]. Although the river of time has flowed for thousands of years from that era, and the level of human science and technology is no longer what it used to be, denying the true nature of the crisis or refusing to take appropriate actions may still cause our civilization to collapse. The leaders of many countries and the United Nations have recognized this. At the United Nations Climate Summit in September 2019, 77 countries and more than 100 cities pledged to achieve net zero carbon emissions by 2050. In September 2020, Chinese President Xi Jinping announced in his speech at the general debate of the 75th United Nations General Assembly that China’s carbon dioxide emissions will reach a peak by 2030 and that China will strive to achieve carbon neutrality by 2060. Such an ambitious goal reflects China’s role and responsibility as a responsible major country on environmental protection and climate change issues.

Since the implementation of economic reforms and the opening-up of policies in 1978, China’s economy has transformed into a market economy. With the rapid economic growth and the acceleration of urbanization and industrialization, it will inevitably be intricately intertwined with climate change. China has maintained a high level of economic growth since the 1980s, and at the same time, carbon dioxide emissions are also increasing [21]. According to the calculation results of the Energy Consumption and Emissions of Buildings study in China by the Building Energy Research Center of Tsinghua University, China’s building construction and operation energy accounted for 33% of the total energy consumption of the whole society in 2019, which is close to the global proportion. However, China’s building construction accounts for 11% of the entire society’s energy consumption, which is higher than the global proportion of 5% [22]. The vast majority of energy consumption and greenhouse gas emissions related to the building sector occur in the two stages of building, construction and operation [23]. Building operation accounts for 23% of China’s total energy consumption, which is lower than the global average. In the future, with economic and social development and the improvement of living standards, the proportion of building energy used in the entire society will continue to grow. From the perspective of carbon dioxide emissions, in 2019, China’s building construction and operation related carbon dioxide emissions accounted for about 38% of China’s total carbon dioxide emissions, of which building construction accounted for 16%, and building operation accounted for 22% [22].

China’s building area is as high as 400–600 billion square meters, but according to the survey, less than 10% of the buildings are energy efficient and have considerable potential for building renovation [24]. Due to the long use of buildings, most buildings will continue to be used for the next 50–100 years [25,26]. Studies have shown that the renewal of existing buildings leads to building upgrades, which are more capable or at least better than before in environmental, social, and economic aspects after renewal [27]. Therefore, building renovation is an effective way to reduce energy consumption. Building renovation usually aims to upgrade buildings through the implementation of green technologies and environmentally friendly materials [28]. In the past few decades, it has been widely recognized that building renovation is an opportunity to cope with the current challenges of primary energy reduction and global warming [29]. In this article, the term “renovation” is used as a general term for improving the performance of existing buildings, “decision makers” mainly refer to professional teams with knowledge and experience in the field of architectural design and construction, and “sustainable renewal” refers to a holistic plan that strikes a balance between the environment, society and economy.

As the problem of dealing with high carbon emissions in stock buildings has gradually become a worldwide problem, a large number of developed and developing countries have conducted extensive research on building renovation. Recently, the number of papers related to building renovation has increased rapidly. Related research includes the definition and scope of building renovation, renovation technical methods, building renovation stakeholders, and the driving forces and obstacles that drive renovation. Existing research on building renovation and renovation methods starts from the demand side and the supply side, respectively. The topics of intensive research include heating and cooling demand-reduction technology, energy-saving equipment and low energy-consumption research, research on energy-usage patterns of building users, renewable energy technology, and electrical systems transformation. In addition, a large number of literature reviews on building renovation have been published; Pombo et al. reviewed the research on housing renovation and discussed methods for evaluating energy-saving measures [30]. In order to find the best restoration solutions and the actual improvement potential of housing renovation, research results emphasized the need to apply life cycle methods. Nielsen and others reviewed the existing literature on decision support tools applicable to the pre-design and design phases of retrofit projects, with the purpose of providing a state-of-the-art overview and suggestions on the future development of decision support tools in retrofit projects, including retrofit aspects of multiple buildings [31]. Sobolewski studied the contradiction between theoretical savings potential, legislator requirements, pollutants in and around existing buildings, and the actual waste of resources [32]. Jagarajan and others critically reviewed the existing literature on green restoration and identified contemporary research trends [33]. Their study emphasized the need to determine the critical success factors (CSF) for the successful implementation of green restoration projects, and believed that the determination of success factors should be based on interest problems faced by stakeholders.

Although there is a substantial quantity of research on building renovation technology in the market, when choosing a specific implementation plan and renovation technology for a specific project, few documents comprehensively consider a variety of influencing factors. The decision-making motivation of buildings with different functions and at different stages in deciding whether to carry out building renovation is also restricted by many factors. The final solution is often not the best one in terms of economic and environmental benefits, but is just a compromise between a series of regulatory or non-regulatory factors, and similar factors such as laws, regulations, technology standards, economic conditions, energy, stakeholders, the surrounding environment, etc. The existing research is either limited to a few subjects or from a comprehensive macro perspective. Even though there are a few studies that include a wide range of analysis, they only look at and analyze different factors in isolation. A large number of industry practices have shown that using language far from the actual format of the building to carry out integration lacks practical significance. This will hinder the systematic planning of the top-level design of the building renovation project, which will affect the implementation of the subsequent phases and the achievement of the renovation goals.

What differentiates this paper from the abovementioned works is that it first describes in detail the existing building renovation research and the application of new technologies, and then takes into account the integration of building characteristics (energy, economy, technology and regulations) and discusses the three parties that may play a decisive role in promoting the low-carbon building renovation agenda. The so-called three parties basically include Party A (investors, owners, relevant community groups), Party B (designers, constructors, suppliers), and the construction authority. It does not rule out that the identities of the three parties may be exchanged in some special circumstances.

## 2. Data and Method

### 2.1. Data Source

After comparing the databases through a preliminary search, we finally decided to use the data in the core database of Web of Science as the data in this study. By using a combination of search phrases related to building renovation, combined with the three search fields of title, abstract, and keywords, the relevant articles were searched. By reading the article titles and abstracts, the retrieved articles are further accurately screened manually, and the documents that are not related to the building renovation are eliminated.

We also searched in CNKI with “building renovation” as the keyword, and received nine related articles. We also received relevant opinions from professionals working in the Chinese construction industry. These files are not included in this article.

### 2.2. Research Methods

**Figure 1:**
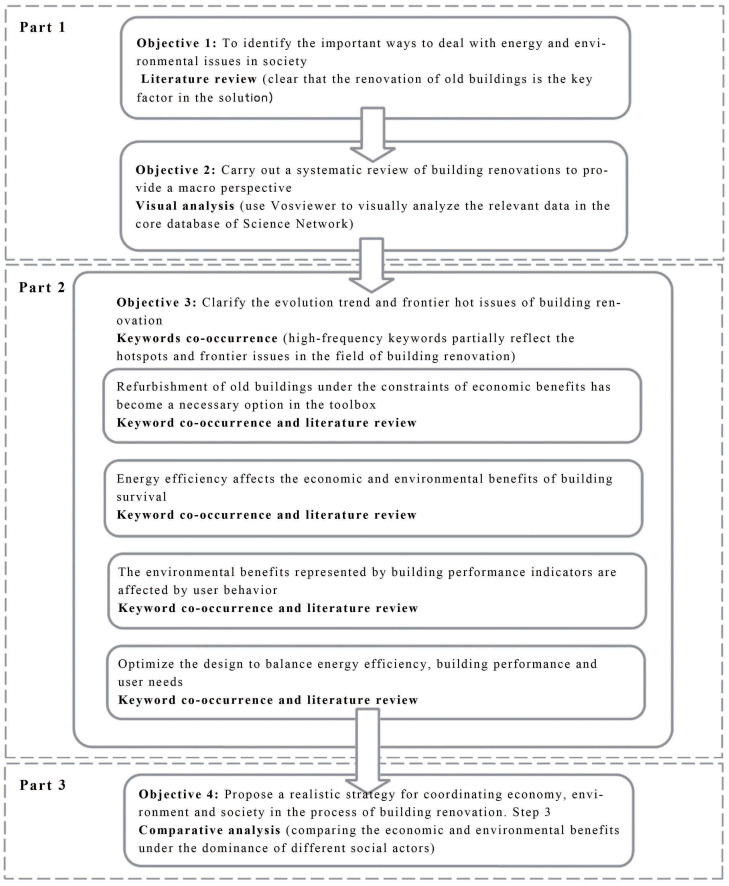
Research process.

VOSviewer is a visual science and technology document analysis software based on the JAVA platform, which can be used to make graphics to visualize document network data. It can detect and visualize the development trend of scientific disciplines and important changes in the development process. This article uses VOSviewer (1.1.16) to establish a national and journal cooperation network. In addition, a keyword co-occurrence network was also carried out. The research process is shown in Figure 1.

## 3. Visualization Analysis

### 3.1. Quantity of Papers’ Trends

In addition to the environmental problems and energy crisis caused by the climate crisis, the existing old buildings in various countries around the world consume huge amounts of energy, and at the same time, there are problems such as lack of necessary maintenance, poor quality of the surrounding environment, and imperfect living facilities. The issue of the renovation of existing buildings is becoming more and more prominent. Taking residential buildings as an example, the old urban area, as the former center of the city, plays a vital role in the economic development of the city. However, with the expansion and development of the city, the economic vitality of the new urban area continues to be highlighted, attracting people to live there. The existing residential buildings in the old city are dilapidated in appearance and imperfect in function, which severely restricts the development of the old city.

In addition, the construction standards and related technologies of many existing buildings in the construction stage are not yet mature. In many residential buildings that have existed for a long time, there are problems such as poor indoor sound insulation, unreasonable spatial layout, and low lighting efficiency, which affect the residents’ lives. Due to the existence of reasons such as the above, countries around the world have conducted a lot of research on building renovation, and research papers have increased rapidly. These studies include updated technologies and methods, stakeholder dynamics, laws and regulations, etc. Abdul Hamid and others researched 234 references related to the renovation of multifamily buildings in temperate climates [34]. The article pointed out that before 2000, only 5 references were considered relevant to its research, but from 2009 to 2013, the number of available references increased from 7 to 38. Starting in 2013, the number of references per year have been no less than 30 articles. The research of [35] and others also found related trends [36]. The number of references in this sustainable building renovation research has rapidly increased from 30 in 2011 to 100. After 2015, the number of publications each year has exceeded 100, indicating that building renovation is becoming more and more important.

**Figure 2:**
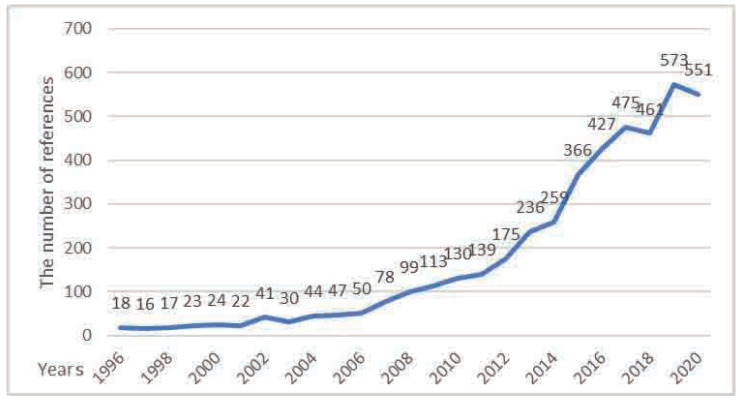
Trends in the number of documents.

In this paper, we searched for building renovation in the core database of Web of Science, and we found that the search results show that related research is in a rapid growth stage in recent years, as shown in Figure 2.

Figure 2 shows that the search results before 2000 remained at about 20 per year, with no obvious fluctuations. From 2001 to 2008, the search results of building renovation approached the hundred digits in an increasing manner year by year, reaching 113 in 2009. The growth rate has also accelerated since 2009, doubling on average every three years, and reached a peak of 573 in 2019. Through manual screening, we selected 1408 of these records The relatively high degree of literature has launched a visual analysis.

### 3.2. Co-Citation of Journals

Journal co-citation analysis is a quantitative research method in bibliometrics and scientific metrology. It has been widely used in many disciplines by domestic and foreign scholars. Through co-citation analysis of journals, journals can be positioned and classified, the core or marginal status of journals in the discipline can be determined, and academic journals can be evaluated.

**Figure 3:**
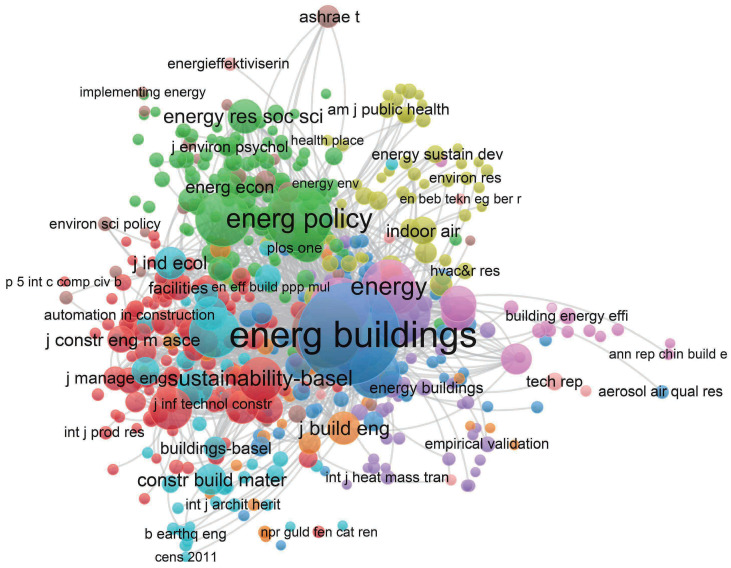
Co-citation of journals.

This article combines the characteristics and theoretical discussion of journals, studies some issues, and seeks to improve and innovate the co-citation analysis methods of journals. When we made the visualization atlas, we selected journals to be cited as the subject of analysis. In order to simplify the analysis, we chose to properly filter the citation frequency of the journal, and finally obtained a co-citation network diagram composed of 616 connected nodes; the result is shown in Figure 3.

**Figure 4:**
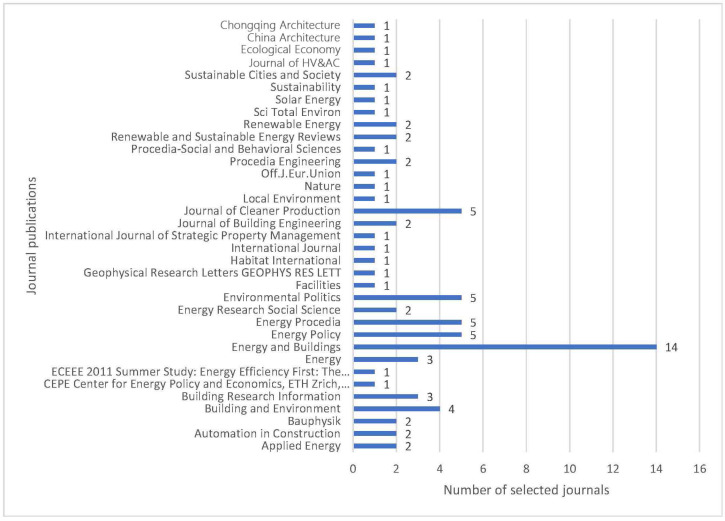
Number of cited publications.

Among them, energy and buildings are the most cited documents. The number of citations is 3.02 times that of the second-place building and environment, 3.63 times that of the third-place energy policy, and 5.01 times that of the fourth-place renewable and sustainable energy reviews. The fifth place is 5.05 times that of applied energy. From the sixth to tenth place are Energy, Journal of Cleaner Production, Building Research and Information, Journal of Building Engineering, and Sustainable Cities and Society. Obviously, energy and construction are at a key position in the research in the field of building renovation. In the following further analysis, this article cited various articles from different publishers, as shown in Figure 4.

### 3.3. Cooperation of Countries

**Figure 5:**
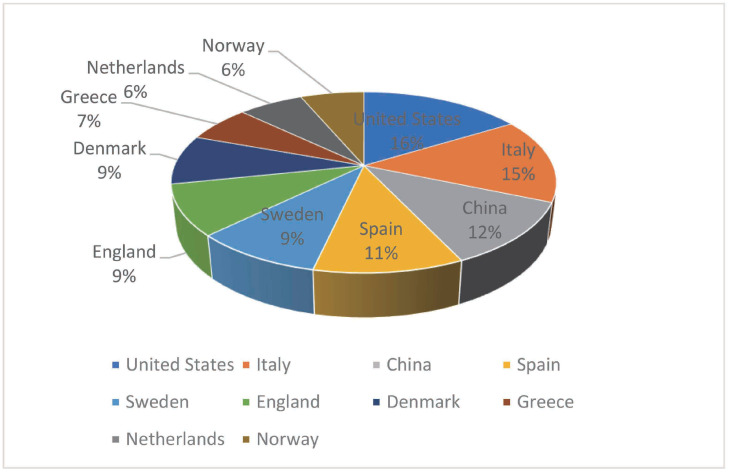
Proportion of country-related papers.

**Figure 6:**
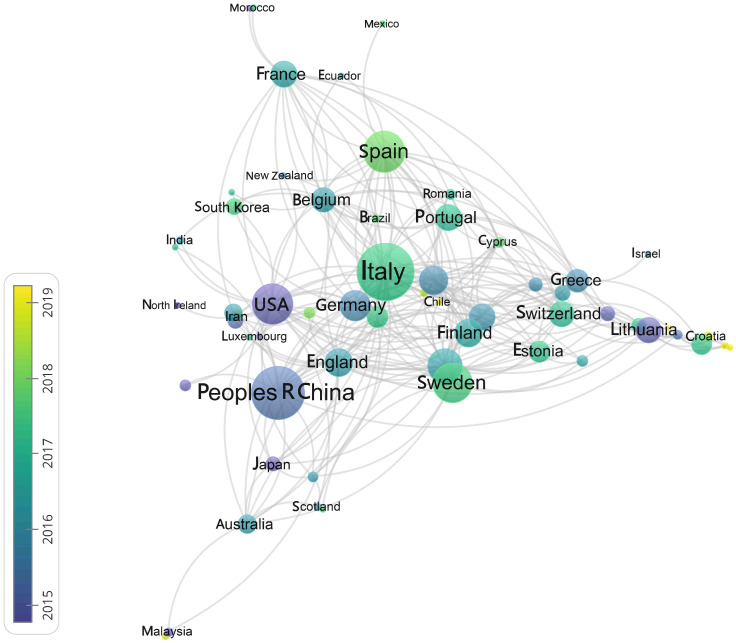
National cooperation of countries.

When we selected countries as the analysis subject, in order to make the analysis more pertinent, we selectively deleted some nodes that did not contribute to the analysis results. In the end, a cooperative network composed of 77 node connections is obtained, and the result is shown in Figure 5 and Figure 6. We found that the number of papers published in Italy, China, the United States, Spain, and Sweden far exceeds that of other countries.

In many European countries, research on building renovation started early. Because 90% of the European building stock was built before 1990, it is estimated that the annual growth rate of new construction in the residential sector is about 1% [37,38]. Because it is critical to evaluate the entire life cycle, it is a challenge to achieve low energy consumption standards while being cost-effective in existing buildings [39]. Historic buildings are a distinctive feature of many cities, towns and villages in Europe, and represent a rich European cultural heritage and a living symbol of diversity.

Due to the increasing shortage of energy resources and the gradual aging of historical buildings, since the 1970s, the United Kingdom has paid more and more attention to the goal of improving energy efficiency and reducing energy demand through renovation and transformation [40,41,42]. Britain’s strength in building protection can be traced back to the 19th century, and has developed rapidly since the 1930s. At that time, the economy was in recession, a large number of important buildings were demolished, and then the bombing of World War II caused immeasurable building damage. Therefore, social groups and the government actively responded to the trend of building protection. Recent surveys show that about 40% of overseas tourists consider heritage sightseeing as their main reason for traveling to the UK, while 53% of Britons travel to historic cities and towns at least once a year through one-day tours and holidays [32]. Since the 1990s, the EU has played an increasingly important role in responding to climate change. At the same time, in order to mitigate climate change by reducing carbon emissions, governments have made political commitments. All sectors of society have begun to realize the benefits of improving the weather resistance of old buildings and reducing the cost of building operation [42,43]. The British “Climate Change Act” promulgated in 2008 made a promise: by 2050, to reduce carbon dioxide emissions by 80%, which makes the United Kingdom a leader in countries that support the concept of low-carbon economy.

Compared with the built environment in Europe and the United Kingdom, China has more modern buildings and a large number of projects under construction, but not all new buildings can meet current energy-saving standards. For example, in 2006, only 38% of new buildings in urban areas met energy-saving standards [44]. Compared with Europe, China’s energy efficiency measures for the built environment started relatively late. The initial attention began in the 1980s, when the first building energy efficiency code-“Civil Building Energy Efficiency Design Standard (Part of Heating Residential Buildings)” was introduced as a national standard in 1986. Since 1986, a series of building energy efficiency codes and standards for different building functions and different building climate zones have been issued to deepen the building energy efficiency goals. However, all these codes mainly guide and influence the design of new buildings, and the scope of application is limited to newly built, renovated, and expanded buildings, and rarely involves old buildings. Most of the building energy consumption still comes from existing buildings [45]. This creates a huge space for subsequent large-scale energy-saving research. The situation in Hong Kong and China is different. More than 89% of the residential buildings here were built before 1998. Most buildings require irregular maintenance due to low performance, accessibility, safety, poor indoor air quality, etc. These buildings will have to be renovated due to poor performance. The research of Chan et al. conducted a coherent review and research on the relevant influencing factors of building renovation in Hong Kong, and provided useful references for local governments and other countries and regions [46,47,48,49].

Germany has become the leader in theoretical and technical research on the renewal of existing buildings, and has also achieved outstanding results in the practice of building energy-saving renewal [50,51,52]. The German government has promulgated a series of laws and regulations to promote energy-saving buildings. Driven by policies, Germany pays attention to energy saving in building renewal. Its energy-saving and renewal technology is worth learning from the experience of implementing incentive policies for the renewal of existing buildings. The time when the United States began to study building renovations was almost at the same time period as Germany. Goldman et al. in 1988 analyzed the renovation of American buildings from the perspectives of energy saving, cost, and economy [53].

Approximately one-third of buildings in Sweden are more than 50 years old [37,54,55]. Currently, the total number of buildings with some form of heritage protection in Sweden is not exact, although this number is estimated to be between 8000 and 30,000 [56,57,58]. In 2017, the Swedish government pledged to achieve a long-term goal until 2045, when the country intends to achieve net zero greenhouse gas emissions [59]. Norway has also been committed to creating a power certificate market since 2012 in order to increase the generation of renewable energy [60].

In addition, Italian research has also made a great contribution to the field of building renovation. Hong and others introduced the latest developments and current obstacles in simulating occupant behavior and quantifying its impact on building energy use [61]. Ascione and others put forward a deepening on the potential of phase change materials (PCM) in reducing cooling load of building envelope, considering the building cooling demand in five Mediterranean climates: Ankara (Turkey), Athens (Greece), Naples (Italy), Marseille (France), and Seville (Spain) [62]. In order to select effective renovation measures and quantify the energy-saving potential of the existing building stock, “reference buildings” should be analyzed; Ballarini and others introduced a method of identifying reference buildings [63].

More than a third of Austria’s final energy consumption is used to provide space heating, domestic hot water, and cooling for residential and service buildings [64,65,66]. The rate of new construction starts is relatively low, accounting for only 1–1.5% of Austria’s annual construction stock [67]. Therefore, the greatest energy-saving potential lies in existing buildings that need to be refurbished. Approximately 75% of Austria’s two million buildings are single-family or two-family houses, which offers great potential for cost savings [68]. In terms of construction period, buildings constructed between 1961 and 1980 have the greatest potential because one third of the entire building stock was built during this period [69].

## 4. Co-Occurrence of Keywords

The analysis method of constructing the keyword co-occurrence network has been deeply applied in the literature analysis and research of various disciplines. Through the cluster analysis of the common content appearing in the article, the current situation and development trend of the discipline research can be revealed. We choose keywords as the analysis subject, and plan to achieve the following three goals by constructing a complex network of building renovations and digging in-depth article content information:

- Sort out similar concepts and clarify the definition, theoretical basis, and technical methods of building renovation;
- Construct a network map, conduct a comprehensive analysis of keywords in related articles, and grasp the research hotspots and trends of architectural renovation from a macro perspective;
- On this basis, look for the deficiencies of existing research and summarize topics that need further research.

**Figure 7:**
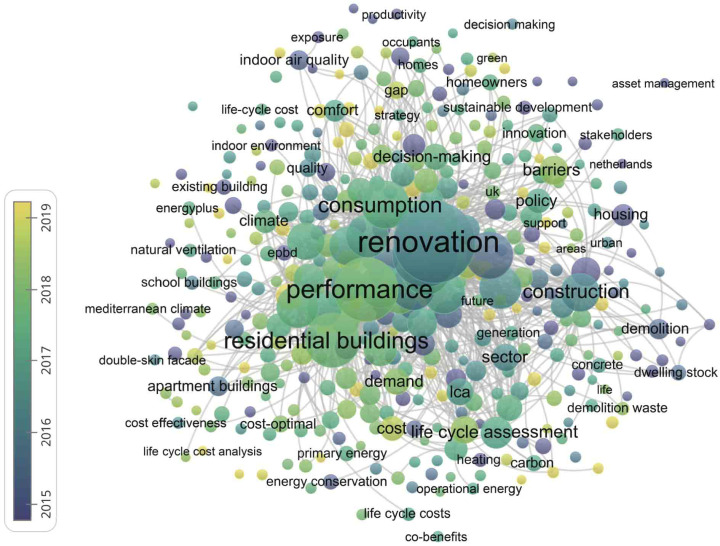
Co-occurrence of keywords.

First, use the relevance of building renovation to screen the documents found in the search, and secondly, appropriately screen the publication year to determine its relevance to building renovation. Due to changes in technology and environment, the conclusion does not match reality, so references to architectural renovation before 2000 will be excluded. Of course, if the relevance is considered high, the references to the architectural renovation theory will be included. As with the previous steps of making the graph, we first select keywords as the analysis subject. In order to make the analysis more targeted, we selectively deleted some nodes that did not help the analysis results, and finally obtained a connection consisting of 406 nodes. The results are shown in Figure 7.

In this map, the high-frequency keywords listed by ranking do reflect some of the hotspots and cutting-edge issues in the field of building renovation, but there is a problem that is difficult to avoid. The high-frequency keywords are either highly repetitive or relatively isolated from each other, and do not meet the purpose of looking at building renovation from a macro perspective.

**Figure 8:**
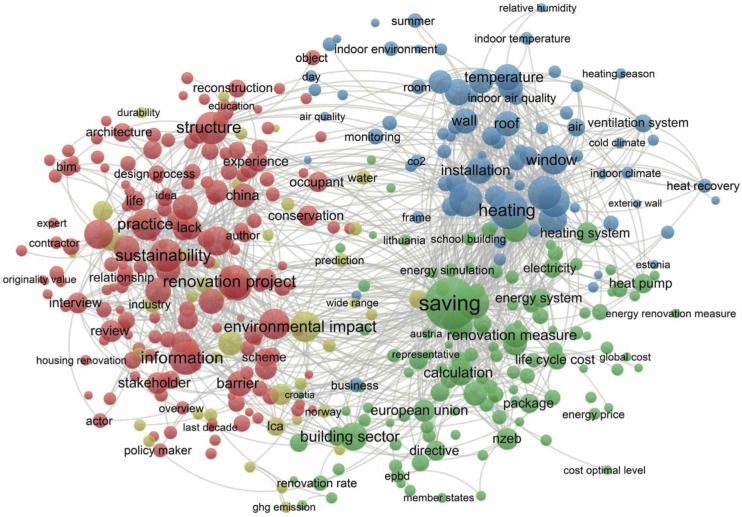
Co-occurrence of professional terms.

In order to understand the network more clearly and accurately describe the research status of building renovation, the overall structure and important node indicators of the keyword network are analyzed. We also chose technical terms as the subject of analysis. After selective deletion and filtering out some nodes that did not help the analysis results, we obtained a network graph consisting of 418 connected nodes. The result is shown in Figure 8.

In this new map, more than ten nodes such as relative humidity, heat recovery, heating season, cost optimal level, ground source heat pump, design methodology, demolition waste, ventilation system, heat demand, and building information modeling are ranked in terms of correlation. Forefront. After combining the keyword co-occurrence map and the term co-occurrence map, we got rid of loose isolated research and improved the correlation between keywords. After a comprehensive analysis of these closely-connected research maps, we can understand building renovation more clearly, accurately describe the research status of building renovation, and conduct accurate analysis of the overall structure and important nodes of the keyword network.

After removing some of the keywords, we believe that the research on building renovation as a whole revolves around the core points of renovation, energy efficiency, performance, residential buildings, consumption, energy, renovation, design, sustainability, and optimization. A systematic literature review was conducted.

## 5. Critical Issues and Their Solutions

The people have always had a need for a better living space. The improvement of energy efficiency can fundamentally optimize the living conditions of old buildings and reduce the carbon emissions generated in the process. The building renovation toolbox has the tools to solve the energy consumption problem of the building alone. Passive and active design can be used to improve energy efficiency and help us eventually reach net-zero buildings. This will also affect the maintenance and improvement of building performance [70,71], limit the upgrading of the living conditions of old buildings, or require high costs to maintain basic livable spaces. Energy efficiency, building performance, and human settlement requirements constitute an inter-influencing relationship here, and they need to be resolved.

### 5.1. Refurbishment of Old Buildings Is a Must

Refurbishment will be replaced by other words in different documents, which may constitute an obstacle to the understanding of this article. Thuvander et al. [72] studied the process and meaning of building renovation and pointed out that change, adaptation, renovation, restoration, renovation, modification, restoration, reconstruction, re-commissioning, modernization, renovation, adjustment, etc., are all approximate expressions of renovation [73,74,75,76]. The scope of changes in buildings can be minor repairs or renovation, with minimal intervention, or major changes to the original buildings. From a scale point of view, the slightest renovation is usually to protect the original building [77], while the most thorough renovation is to clean up, rebuild, or replace the entire building [54,78].

The renovation method of demolishing and rebuilding old buildings will cause a great waste of resources, and demolishing and rebuilding will also destroy the extension of the urban context. Astmarsson et al. [79] believes that building renovation refers to the process of refurbishing or replacing existing parts of a building to improve its performance, either by restoring it to its original state or improving it. However, the refurbishment discussed in this article means renewal, and is not the same as maintaining and replacing old parts with similar new parts. When refurbishing, the building standard is usually upgraded to be closer to the current standard, rather than the standard when the building was originally constructed. The building standards produced have evolved over time and are mostly increasing, so new buildings usually have higher standards than old ones. Energy demand is a prominent example. In the past few decades, the building codes of most countries have gradually become more stringent in terms of reducing building energy consumption. Akadiri et al. [80] stipulates the green renovation of existing buildings as activities aimed at conserving energy resources, improving the living environment, and enhancing the use of functions, etc., to maintain, update, and strengthen existing buildings [81]. The green transformation of existing residential buildings is different from the energy-saving transformation. The energy-saving transformation of existing buildings is a comprehensive transformation of energy consumption systems such as building envelope, air conditioning, heating, ventilation, lighting, power supply and distribution, and hot water supply. The green transformation is more comprehensive, not only focusing on energy conservation, but also including the improvement of the living environment and the enhancement of functions.

### 5.2. Energy Efficiency and the Survival of Old Buildings

The concept of energy efficiency was first proposed after the outbreak of the two world oil crises in the 1970s. Governments of various countries have been deeply affected by the oil crisis. With the purpose of saving fossil energy, they have successively formulated a series of policies and regulations, strengthened research within this framework, and developed energy-saving technologies. In 1979, the World Energy Commission defined “energy saving” as taking all measures that are technically feasible, economically reasonable, and environmentally and socially acceptable to improve the efficiency of energy resource utilization. In 1995, the World Energy Council defined “energy efficiency” as “reducing energy input to provide equivalent energy services”.

As people are concerned about the issue of sustainable development, energy conservation has become a research hotspot [35,82,83,84,85]. Improving energy efficiency is one way to reduce carbon dependence. Compared with other green energy sources such as wind and solar energy, the implementation cost of energy-saving technologies is much lower. Leveled energy costs and life-cycle costs are advanced analysis methods that can be used to evaluate cost-benefit transformation schemes for multiple energy sources [86]. Because building energy consumption accounts for more than 40% of the total energy consumption [87], energy-saving renovation of buildings has attracted widespread attention [88]. Many scholars have studied how to improve the energy efficiency of building renovation [89,90]. A high-efficiency precast concrete element (PCE) system was developed by using construction and demolition waste as raw materials for energy transformation of residential buildings. It was developed in Spain, the Netherlands, and Sweden by covering the walls of old buildings. Research objects using life-cycle assessment and cost methods explore the life-cycle performance of the PCE system in terms of energy conservation, carbon emission reduction, and cost reduction [91]. Through a historical building (a Victorian house in the late 19th century), the energy-saving potential, economic efficiency, and thermal comfort of various passive building renovation measures were evaluated, and the recommended passive renovation combination was finally obtained [56]. Using the method of installing two different PV systems on a 150-year-old castle in Helsingborg, Sweden, using the life-cycle cost (LCC) of the net present value (NPV) method to calculate the maximum power generation. The primary energy generated was compared with the primary energy use of historical buildings to assess the potential of the Nordic historical buildings to be transformed into NetZEB buildings. The final success proved the feasibility of net-zero construction. Some scholars have evaluated the energy-saving efficiency of building renovation [92,93,94].

### 5.3. Building Performance Serves the Needs of Human Settlements

Building performance has always been the focus of researchers. The most mainstream green building evaluation systems in the world today, such as the American Leadership in Energy and Environmental Design (LEED) [95] and the British Institute of Building Research Environmental Assessment Method, have all been in recent years. The most recent version of “Performance” mentioned the keyword, and some also set up a special evaluation path for performance. As one of the three typical green building evaluation systems, Chinese domestic scholars have also begun to pay attention to this field [96,97]. Thermal comfort is one of the important indicators of building performance [98]. Scholars investigated the thermal comfort of apartments (16 apartments) that are generally equipped with heating systems in historical buildings in Polish cities. Their research found that the thermal environment of solid fuel heating is lower than that of centralized gas heating, but the findings of personal thermal feelings and preferences did not confirm these findings. Du et al. [99] tried to develop a general plan for indoor environmental quality assessment. The study demonstrates the potential impact of building renovations on the thermal environment of the occupants, and shows that simply adjusting the indoor temperature can help save energy. In order to achieve the best energy-efficient indoor temperature that meets thermal comfort, Zahid et al. [100] describes an optimization method that combines BIM (building information and modeling) and IoT sensors (Internet of Things). This integration allows the use of geometric and parameter richness of the BIM model and the real-time streaming of environmental data (humidity, temperature, etc.) collected by IoT sensors to optimize indoor thermal comfort. In addition, [101] introduced a method of uncertainty quantification and sensitivity analysis to improve design schemes and building performance in operation. Sensitivity analysis is often used in building performance simulation to help find the main performance factors.

### 5.4. Optimized Design Integrates Energy Efficiency, Performance, and Demand

Design refers to a process from imagination to reality before a building is constructed. According to the construction task, the designer makes a comprehensive vision in advance to solve the problems that exist or may occur during the construction process and the use process. The method and plan of the project are expressed in drawings and documents. However, in the context of architectural renovation, the keyword design is given more content. Different from designing new buildings, the related parties and aspects that need to be considered in the renovation design of existing buildings are more and more complicated [102]. Since the main part of building energy consumption is related to maintaining a suitable indoor climate [103], in order to obtain ways to improve carbon emissions, past research and policy efforts have focused on more energy-efficient buildings and technologies. However, the actual energy consumption of space heating depends not only on the efficiency of the equipment, but also on the usage of the residents, how users adjust the indoor climate, and why they do it [104]. Taking Germany as an example, the heating energy consumption of a family may be six times that of another family of the same heat level [105]. This phenomenon is not unique to Germany. For example, it is also evident in Switzerland [106], France [107], Austria, the Netherlands [108], and Denmark. Kempton et al. [109] describes and analyzes how culture and technology affect the comfort temperature at different times and places. These studies provide details on how people adjust and use their air conditioning systems.

The improvement of energy efficiency can fundamentally optimize the living conditions of old buildings and reduce the carbon emissions generated in the process. The use of passive and active design to improve energy efficiency will also affect the improvement of building performance. Energy efficiency, building performance, and human settlement requirements form an inter-influential relationship here. At the moment, when new technologies and new theories are emerging endlessly, how to grasp the balance between the three requires us to start from the dimension of economic benefits and solve the optimization problem in the design link.

The optimization concept used in construction mainly refers to the use of computers to optimize building performance. The process is to determine the best solution for a given design problem or control problem from a series of alternative schemes based on the selected building performance target using an optimization algorithm [110,111]. Compared to manual trial-and-error optimization, this method can explore more possible design schemes and ensure that the exploration direction is better. Since the building itself is a complex system, its geometric form, envelope structure and many other architectural elements interact, so the overall system needs to be optimized [112], and the various optimization goals are not independent of each other, but have contradictory correlations (such as energy consumption and cost, energy consumption and daylighting, etc.). Optimizing only a single goal may lead to the loss of other goals. Therefore, multiobjective optimization is the preferred method of building performance, which can make the building’s multifaceted performance level to be collaboratively optimized [113]. However, most scholars consider the optimization problem of economy or environment, and only a few scholars consider the optimization problem of economic, environmental and social benefits [31].

Wanzhen et al. [114] considered the particularity of the renovation of existing buildings, from the perspective of market operation, combed the research dynamics and practical characteristics of energy-saving renovation of existing buildings at home and abroad, and on this basis, explored the integration and optimization of multiple subjects and objectives in the renovation of existing buildings. mechanism. Since the research on zero-energy buildings involves many subjects and objects, in order to solve the limitations of traditional optimization methods, in view of the continuous increase in building energy consumption, [115] combines the model with the comprehensive evaluation method and proposes a new zero-energy building. The global multicriteria optimization model of energy-consuming buildings proposes optimal solutions based on the selected decisions, trying to solve the problems of energy saving, solar energy application benefits, and social economy. There are also some researchers exploring the implementation of multiobjective optimization of building renovation through modeling and investigation [116,117,118].

## 6. Evaluation of Economic and Environmental Benefits

**Figure 9:**
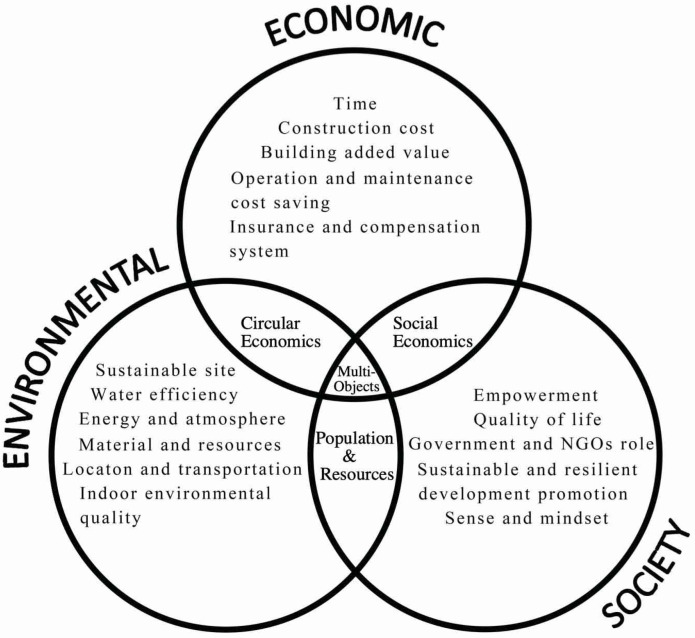
Sustainable retrofit framework.

With the continuous development of society, the phenomena of low building performance, inconvenient traffic in the community, and unreasonable use of the area exhibited by old buildings have become more and more prominent. In addition to the severe situation of social aging, the past mainly used to transform the appearance of the city. Energy-saving and emission-reduction transformation methods can no longer adapt to the current pace of social development, and more humane, scientific, and comfortable transformations are imperative. After years of development for the renovation of old buildings, certain experience has been gained, and some methods of renovation have been summed up. The following uses the sustainable analysis framework in Figure 9 to divide the existing main methods for the renovation of old buildings into the following four types to evaluate their economic and environmental benefits.

### 6.1. Carry out the Renovation of Old Buildings through a Market-Oriented Approach Led by a Small Group of Users

Most of the old buildings were built in the 1980s and 1990s. Due to the technical level and life philosophy at the time, residential buildings generally have small apartment sizes and narrow kitchens and bathrooms, which can no longer meet the needs of modern life. The primary transformation goal is to upgrade and strengthen the key nodes of the building, and add thermal insulation to the external structure of the building if conditions permit. These old buildings have a low floor-area ratio and are mostly low-rise or multistorey buildings. The roof part of the building has a large space for development, and the roof can be added within a reasonable load-bearing range. The advantage of the roof extension is that no additional land resources are required, and the roof extension can be sold to residents of the residential area or rented out by the property company to supplement the funds required for renovation and subsequent maintenance. There are a lot of experience in the renovation of roof extension projects abroad.

However, the current transformation of old residential areas in the form of collective and market cooperation is limited to the insulation and reinforcement of the exterior walls of the building, and the indoor transformation is limited to the replacement of old pipelines, which cannot solve the defects of the house type. It only improves the performance of the building, plays a role in appropriately reducing energy consumption, and cannot effectively respond to people’s demand for livable building space. In summary, the economic benefits of this program are not obvious, and the environmental benefits are limited.

### 6.2. Transformation Led by Large User Groups

This approach is mostly used in the transformation of villages in cities. The village-in-city refers to the villages that were surrounded by urban planning and construction by covering some villages that were close to the city in the past in the process of rapid urban development. This type of transformation is mostly carried out by collectively establishing economic development companies, and the government is responsible for collaboration or supporting policies. The strategies in the toolbox include: collectively making full use of the free space as the starting plot for turnover, building new apartment-style villages, resettling the original residents in the village, and demolishing the old residential areas; the government will renovate the old residential areas at the same time. Similarly, supporting facilities and policy support, the government prepares detailed plans for the renovation of old residential areas, conducts guidance on construction, collectively provides funds for renovation and renovation, and at the same time advocates public participation and respects the wishes of the original residents.

In the case of rebuilding old residential areas led by collectives, a certain proportion of the projects were unfinished due to the lack of strength of the initiators and the shortage of funds for the rebuilding projects. In addition, collectively-led renovation plans tend to lack compatibility with subsequent technological developments, which will weaken the sustainable development potential of buildings. Most of the renovations are only small-scale ways of greening the environment and adding residents’ fitness equipment, and cannot fundamentally solve the chaotic environment of the community and the problem of low order and low building performance. To sum up, the economic situation of the plan is poor, unfinished projects are prone to occur, and the improvement of the living environment is limited, and it cannot achieve the purpose of reducing energy consumption, and may even have a counterproductive effect.

### 6.3. Transformation Carried out by the Government with the Help of the Market and the Collective Cooperation of Users

In this method, the government is generally only responsible for the work of compensation for demolition and land consolidation in the early stage, or in the later stage for policy guidance and standardization of market behavior. The main process is mostly through the introduction of social funds for market operation. Specifically, it includes the following: through the combination of government-led, public participation, and market-oriented operation, the standardization of the transformation process, the transparency of the operation method, the establishment of supervision channels, the complete marketization of the transformation process, and the government’s investment guarantee funds can be realized through the transitional resettlement of residents;the government and developers jointly carry out demolition, land consolidation, and reconstruction, and introduce market funds to realize ecological and industrialization of residential areas; the government actively adjusts the function of land use, introduces social funds to participate in the preliminary land preparation, and jointly develops land operation and management, rationally formulates development intensity, balances the benefits of reconstruction, and reduces the difficulty of reconstruction of old residential areas.

Under the strong leadership of the market, most of the enterprise cooperative development and transformation projects are defined as gradual small-scale renewal; the scale is in a moderate position, and there will be no large-scale radical urban transformation. The detours taken by developed countries on the road to urban renewal indicate that the large-scale methods adopted for the transformation of old cities are often entangled with huge economic interests, and public interests are completely ignored. This is harmful to the interests of urban residents and destroys the historical context; the weakening of the traditional cultural value of the city has a great impact. The active cooperation of the government can provide the required legal and regulatory environment; in the current rapid technological iteration, it can provide reasonable laws and regulations to reduce the resistance to the implementation of the transformation technology. Increasing the practicability of technology has high practical significance. In summary, while ensuring the effective scale of building performance and energy efficiency improvement, the program can effectively achieve the benign development of the building renovation project market, and greatly improve the economic and environmental benefits at the same time.

### 6.4. Reconstruction and Expansion of the Community under the Leadership of the Government

Many cities are currently renovating old buildings under the leadership of the government. In addition to being responsible for compensation for demolition and relocation, the government also conducts related land consolidation and usually takes the lead in the form of capital investment. The main tools in the toolbox are: the government uses the land in the old residential area as the basis for investment in infrastructure and public service facilities, so as to meet the needs of the regional carrying capacity; reconstruction of old urban land that has lost value in old residential areas that meet the requirements of urban development, and provide reconstruction compensation and living security compensation for residents without houses due to the transformation; or the government’s unified acquisition of land for old residential areas that are included in urban built-up areas. To provide comprehensive social security for the deprived residents, at the same time, the government is responsible for formulating a special protection plan to keep the form, color, number of floors and scale of the new building in harmony with the historic buildings in the old residential area. The government usually sets reasonable compensation standards, and guarantees the resettlement of the demolished households, and finally realizes the goal of alleviating the population pressure in the old residential area and improving the quality of life.

**Figure 10:**
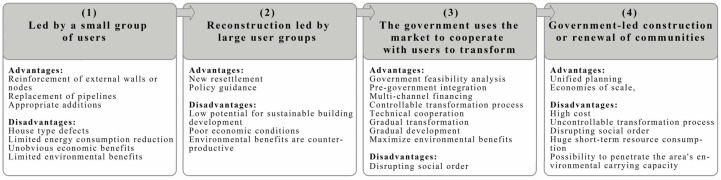
Comparative analysis diagram.

Real cases have proved that the large-scale demolition and reconstruction methods completely led by the government are too costly. In this case, the later urban construction will adopt higher standards to obtain better returns, which cannot be explained. Residents of all strata in the residential area provide a low-cost living environment. With large-scale renovation and expansion the government cannot reasonably control the impact of the renovation project. The renovation process will seriously disrupt the normal living order of residents. Large-scale demolition of old buildings takes away the concentrated memory of a large number of groups, and can easily trigger unpredictable social group reactions. Moreover, large-scale renovation projects with quick success and instant profit may not only fail to achieve the goals of reducing energy consumption and improving building performance, but will generate a large amount of energy and resource consumption in a short period of time, breaking through the environmental carrying capacity in a short time, and are often related to reducing building carbon. The concept of emissions runs counter to this. In summary, the comparative analysis results are shown in Figure 10.

## 7. Conclusions

### 7.1. Economic Barriers

The ability of housing-owning households to finance transformation investments is an often overlooked obstacle to achieving long-term energy and climate goals, especially due to the lack of quantitative evaluation of this financial barrier in the literature. It is estimated that in the Flemish region of Belgium, for example, about half of European households who own houses cannot finance the necessary renovations [119]. This finding applies whether they are financing and performing refurbishment once, or it is scattered over the next few years. Half of the households were able to fund comprehensive reforms but decided not to do so. Considering the potential impact of the COVID-19 crisis on the research results, Albrecht and others found that the proportion of households facing the most serious shortage in financing capacity increased by about 8%. In this case, the general financial environment limits the demand for building renovation business [120,121], and customers usually prioritize project cost, time scale, functionality, and aesthetics [122]. Therefore, there is an obvious dilemma.

### 7.2. Policy Barriers

The main workload of implementing the building renovation still falls on the architect group. However, the architect group is not willing to use unfamiliar materials, and the sustainability assessment of materials is often not as influential as the personal knowledge and past experience of the project team. This is mainly caused by the risk aversion and litigation culture that prevails throughout the industry. The contract structure and procurement route have to a large extent led to a popular “construction and defense” attitude.

Since the traditionally separated construction process involves multiple parties, it will weaken any individual’s ability to make overall project decisions, requiring contract support and guarantees. The contract structure often hinders the effective integration of the design team and the supply chain. Construction professionals usually rely on case studies to evaluate new products. This aversion to innovation is reinforced by the “clearly defined relationship based on contractual obligations”, which restricts inter-firm relationships and information sharing, and strengthens building developers; the designer, the user hierarchy, and the user’s power are lacking. The financial environment, design culture, and contract laws and regulations constrain each other, forming a vicious circle, which has become an obstacle to the mass promotion of low-carbon construction projects.

It is generally believed that government participation is the most basic and effective way to make changes. According to research, market-based incentive programs are effective. However, the government lacks the political will to introduce additional supervision, worrying that this might be seen as another costly “unnecessary bureaucracy” in an already “overburdened” industry.

### 7.3. Design Obstacles

Designers are now faced with an obvious problem: to provide opportunities to interact with buildings and their systems while taking into account the possibility of occupants using them inappropriately. An important feature of the interaction between people and buildings is that occupants in the building have rich and diverse behaviors, which are not easy to analyze and control by simple tasks, and the results of use are difficult to predict. In many interactions between occupants and building controls, similar and conventional unpredictable errors may occur, which leads to performance gaps that are often found and expected during building performance evaluation. In the real world, the addition of automatic control does not always achieve the expected savings, because the controller may change their behavior to deal with them. For example, occupants opening windows instead of adjusting the air conditioner in response to perceived overheating is generally not an energy-saving response. This highlights that if occupants are prone to making routine mistakes when interacting with them, it is futile to integrate increasingly complex technologies in architectural design. Designers often claim that the problem is not in the design of the system, but in the wrong operation of the system by people. However, it is important that the system is created to allow users to perform the necessary functions. If most users cannot easily and consistently operate the system to achieve the desired purpose, then the design will fail. This situation has led designers to regard the occupants as a public hazard and believe that their ability to interact with architectural services should be restricted. Restricting occupants’ control of their environment will have a negative impact on health and satisfaction, and affect energy consumption to a certain extent.

## 8. Proposals and Future Directions

This article provides a comprehensive review of the great potential of green refurbished buildings to create substantial carbon emissions reductions. Including passive and active design, it describes different technologies such as renewable energy (solar energy, wind energy, geothermal energy, biomass energy) utilization technology, building envelope design, and cooling and heating techniques. With the increasing willingness of society to reduce carbon emissions, these technologies and methods will be used and diffused by more practical projects. Therefore, forward-looking municipalities, companies, and building owners are likely to be the beneficiaries of these technologies in the near future. Through the analysis of the challenges in the process of green renovation of existing buildings, it is concluded that a reasonable market mechanism and financial incentive mechanism to encourage technological innovation and renovation and prolong life are inseparable from the government’s renewal of laws and regulations and the government’s promotion of the reform of the construction financial market. Replicable, affordable, and sustainable green building renovation needs to be promoted in the open market to change the high carbon emission status of a large number of old buildings.

The renovation design of old buildings can be combined with the application of new technologies, which can effectively summarize the low-carbon target-oriented optimization design methods for the renovation of old buildings. For example, the research on low carbon building renovation design of BIM technology, through BIM energy simulation technology, explores the impact of external wall heat transfer coefficient, insulation layer thickness, external window heat transfer coefficient, external window shading coefficient, etc. on building energy consumption and carbon emissions, and completes the assessment of carbon footprint, energy consumption, energy balance, etc. in the building renovation plan stage.

## Data Availability

Not applicable.
